# Emergency nurses’ attitudes towards the concept of witnessed resuscitation

**DOI:** 10.1590/1518-8345.1382.3055

**Published:** 2018-09-06

**Authors:** Ana Laura García-Martínez, Cristóbal Meseguer-Liza

**Affiliations:** 1 PhD, Researcher, Universidad de Murcia, Murcia, Spain.; 2 PhD, Assistant Professor, Universidad de Murcia, Murcia, Spain.

**Keywords:** Cardiopulmonary Resuscitation, Heart Arrest, Attitude of Health Personnel, Emergency Nursing, Family Relations

## Abstract

**Objective:**

to review the most relevant evidence on the nurses’ attitudes towards witnessed resuscitation, in the inpatient and out-of-hospital spheres.

**Method:**

integrative literature review, covering the period from 2008 till 2015, using the databases PubMed, Lilacs and SciELO; in Spanish, English and Portuguese. The pediatric context was excluded from the study.

**Results:**

the synthesis of the data resulted in the inclusion of 10 articles, categorized as: positive attitudes and negative attitudes.

**Conclusions:**

discrepancies exist among the nurses from different contexts and geographical regions towards the concept; protocols need to be established for this situation, in view of the advantages evidenced in the literature, for the nursing professionals as well as the relatives. Witnessed resuscitation can represent an opportunity to understand and cope with the rational and irrational in the situation in a shared manner, as well as mitigate or dignify the mourning.

## Introduction

Research on the family’s presence during resuscitation maneuvers^1^ - in the pre-hospital context, defined as the presence and participation of one or more family members in the patient care area, in a place that enables them to have visual and/or physical contact with the patient - started in the 1980’s in the hospital context, particularly at the Foote Hospital in Jackson, Michigan (USA). At that time, the procedure and the traditional medical attitude to the patient’s relatives were questioned, after relatives had requested to be present on two occasions.

As a general standard, at countless inpatient and out-of-hospital services, the professionals try to distance the relatives from the victims of a cardiorespiratory arrest, with a view to avoiding that they hinder the professionals during the application of cardiopulmonary resuscitation techniques. Excluding the relatives is justified under the premise that the invasive procedures and the aggression during the cardiopulmonary resuscitation can provoke stress in the family members and that their presence could compromise the performance of the medical team^2^.

The literature review, however, presents contradictory results concerning the meaning of the concept of family presence during the resuscitation maneuvers and the nursing professionals’ attitudes, adding positive and negative opinions and provoking a continuous debate.

The objective in this study is to review the most relevant evidence on the nurses’ attitudes towards the presence of relatives during cardiopulmonary resuscitation maneuvers inpatient and out-of-hospital services. The idea to have a family member present during the cardiopulmonary resuscitation is supported^3^ and underwritten by different international organizations, such as the Emergency Nurses Association (ENA), the American Heart Association (AHA) and the European Resuscitation Council^4^ ^-^ ^5^.

The lack of protocols on the witnessed resuscitation concept arouses controversies about ethical-care issues deriving from health practice.

## Method

An integrative literature review was undertaken, covering the period from 2008 to 2015. As for the databases related to the health sciences, Pubmed-Medline, Lilacs and Scielo were used. The bibliographic search in the databases was based on the following descriptors and/or key words: *Cardiopulmonary Resuscitation*, *Heart Arrest*, *Attitude of Health Personnel*, *Emergency Nursing*, *Family presence*/*Witness*; in Spanish, English and Portuguese; the following inclusion criteria were used: presence of relatives during the cardiopulmonary resuscitation maneuvers; application of invasive techniques that might be necessary in the procedure and inpatient and out-of-hospital emergency services for adults. Both the qualitative and quantitative method were included in the review.

Therefore, articles related to the pediatric context and articles referring to critical and intensive care services were excluded.

To facilitate the research process, the following guiding question was formulated: Which are the attitudes of emergency nurses to the concept of witnessed resuscitation?

In view of the lack of methodological uniformity for the integrative reviews, in order to analyze the documents, the methodological structure of the systematic review^6^ was used for support, which consisted in the reduction, visualization, comparison, conclusion and verification of the data. In the first phase of data reduction, the categories are identified, which facilitates the analysis; in the visualization phase, the information from the studies was registered; in the comparison of the data, the similarities and differences among the findings were verified; and in the conclusion, the main elements were summarized.

**Figure 1:**
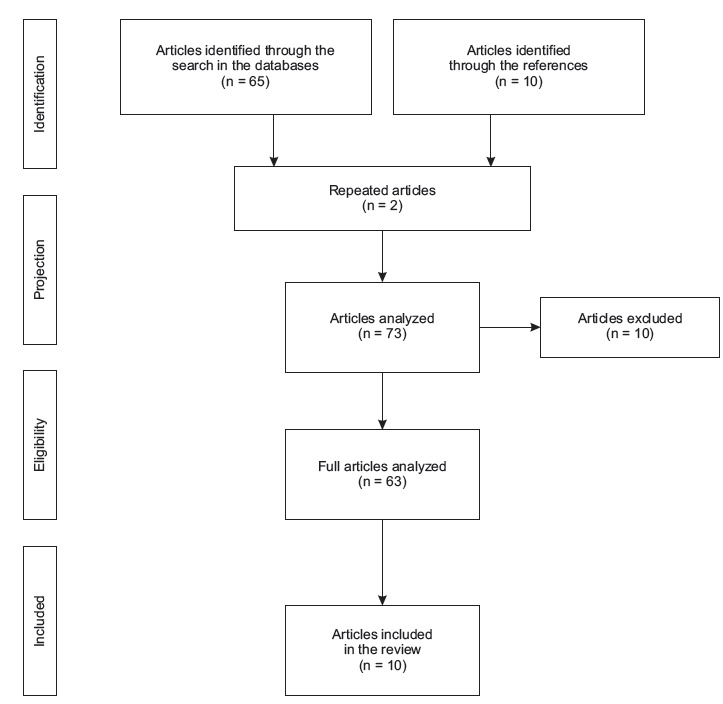
Selection process of the studies in the databases

Among the 20 final articles that could be included in the review, ten were analyzed in accordance with the criteria of relevance and pertinence, including this total in Figure 1. The extent of the document obliged us to give preference to articles that represented the nurses’ attitudes to the concept of witnessed resuscitation with higher quality.

Besides the impact factor of the journal from which the article was taken, the following criteria were adopted: surprising results; theoretical and practical importance; new and interesting ideas; new framework; internal validity: use of appropriate design and method; external validity; the presented results and/or theory are generalizable; sufficient description of the method and procedure for other researchers to replicate them; theoretical or practical results with a high degree of implementation; theoretical or practical results useful to society; and clear specification of the type of study. In addition, the intention was to create a synthetic document, for which those articles were selected that truly offer a determining contribution.

Concerning the limitations of the study, it is highlighting that, given the novel nature of the theme, after applying the filters established, the number of records was limited. This justifies the need to expand the research in this field.

## Results

The ten articles included in the review produced two subcategories related to the attitudes and emotions of the nursing professionals about the concept witnessed resuscitation. It should be highlighted that most of the articles analyzed come from English-language literature, 92 % being located in the database Web of Science, 7 % in Lilacs and 1 % in Scielo.

**Figure 2:**
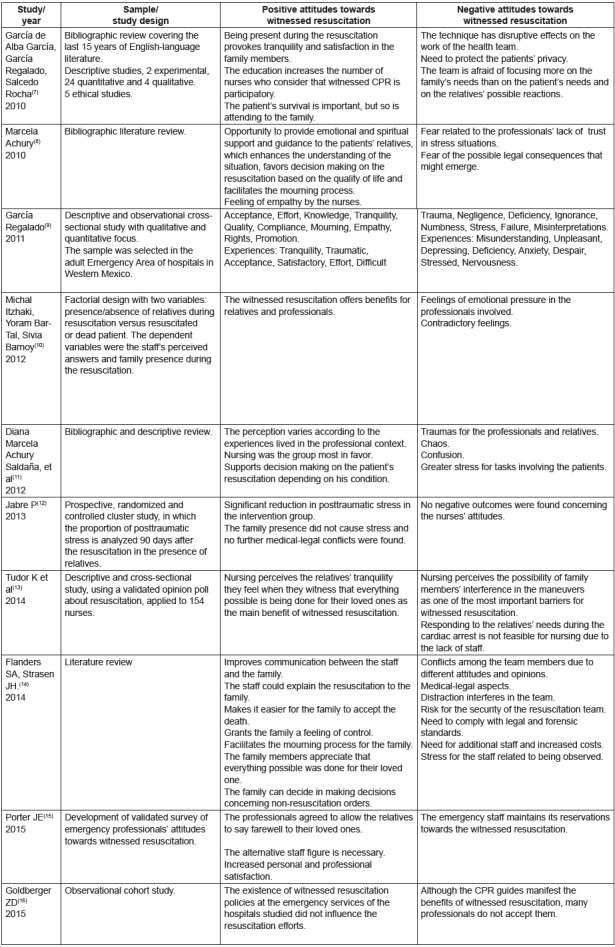
Most relevant results concerning witnessed resuscitation

Figure 2 analyzes the most relevant articles in the integrative review and their most significant results, from which two categories are derived on the emergency nurses’ attitudes towards the concept of witnessed resuscitation.

## Discussion

The main result this integrative review contributes relates to the controversies among the resuscitation team members regarding the concept of witnessed resuscitation; that is so although the most recent CPR guides, such as the guides of the *American Heart Association* and the *European Resuscitation Council* ^17^, identify the benefits of applying family presence policies during the resuscitation maneuvers, respecting the cultural and social values of the family members and even of the professionals involved.

The concept generates an ongoing debate among the nurses, with great variation in the perceived risks or benefits of the family members’ presence^18^. Furthermore, in this study, the findings suggest that the perceptions of the nurses who granted the relatives the opportunity to witness the resuscitation differ from the perceptions of those who did not; the former perceive greater benefits and the emergency nurses are the most willing to invite the relatives to witness the resuscitation.

Qualitative research on witnessed resuscitation could reveal more concrete aspects than quantitative studies and reveal specific benefits of the family’s presence during the resuscitation event. Recent qualitative studies^19^ highlight the need for the nursing professionals to support the family members when deciding on whether to witness the resuscitation or not.

The literature review shows us results on the existing relation between the nature of the background experience in witnessed resuscitation situations and their attitudes^20^. The nurses who informed on positive experiences had significantly more favorable attitudes when considering the benefits of witnessed resuscitation: less fear of negative consequences and less personal and organizational barriers.

Less articles were found in Spanish. Among these, a qualitative study^21^ should be highlighted though, in which the in-depth interviews revealed three main themes concerning the attitudes of nurses active in witnessed resuscitation: unsafe practice, empathetic experience and necessary practice. The positive attitudes were: wellbeing, pride, consolation, conciliation, responsibility, experience, tranquility and acknowledgement. Negative attitudes were: sadness, impotence, stress, nervousness, insecurity, logistics, pressure, anxiety, anguish, lack of control and pressure.

In short, the nurses’ attitudes in executing witnessed resuscitation maneuvers before relatives are dynamically concentrated in these main themes during the maneuvers through different emotions, conducts and behaviors, distributed between signifying a negative experience that affects the professional wellbeing or a positive experience leading to the resilience of nursing. A team member needs to support the relatives during the resuscitation maneuvers.

In the end, the literature review shows us important benefits of witnessed resuscitation. In addition, most of the nurses consider that, when accomplishing the resuscitation maneuvers in the presence of relatives, their stress, lack of control and emotional tension neither provoke interference nor hamper the nurse’s work; thus, the family’s presence is valued, considering it as beneficial, stress reducing and facilitating the mourning process, which is a right of the patient and the family.

## Conclusion

The literature review shows multiple studies on the emergency nurses’ attitudes, in which we can distinguish two categories that contain the different attitudes the witnessed resuscitation event produces; these can be summarized as follows: 

- Positive attitudes: tranquility, empathy, safety, pride and facilitating the mourning process. The witnessed resuscitation can be an opportunity to understand and confront the rational and irrational in the situation in a shared manner and to mitigate or dignify the mourning.

- Negative attitudes: stress, fear, impotence and mainly the feeling that the family members can make the accomplishment of the resuscitation maneuvers more difficult.

The evidence shows the multiple benefits of witnessed resuscitation, as being present during the resuscitation provokes tranquility and satisfaction in the relatives, as well as the need to actively prepare the nurses to enhance their confidence in view of the management of the concept, which justifies the need to establish protocols for this situation. The establishment of agreed protocols would be recommended, which identify when, how and to whom the witnessed resuscitation should be offered.

In the light of the review, it was verified that witnessed resuscitation is a controversial and current team in intra and out-of-hospital emergency care, leaving many unanswered questions nowadays. In that sense, the health professionals are immersed in a great emotional debate in their daily work, when they need to perform violent, invasive techniques or maneuvers in the presence of family members, under the emotional burden this action provokes. Therefore, discrepancies emerge among the nurses from different contexts and geographical zones with regard to the concept of witnessed resuscitation.

It should be kept in mind that the extra-hospital environment is hostile, in which we can often feel unprotected, for example, when a patient is resuscitated at his own home, in the presence of the family, listening to what the team comments; this moment requires great physical and mental effort towards the victim of the cardiac arrest. Nevertheless, it should never be forgotten that the family will need our support; hence, a holistic and comprehensive focus needs to be established in view of the family’s needs.

Concerning the implications of the findings for professional practice, the knowledge and study of the witnessed resuscitation concept and the attitudes observed in the nurses could consolidate its promotion and use, due to the different benefits of this technique the studies evidenced, although some controversies were evidenced; we found a higher value of the nursing professionals’ attitudes. In fact, two branches of the implications were visible: an immediate implication that reflects the safety in practice and the defense of the patient’s values (principle of autonomy), and another indirect implication, which is related to the consequences for the relatives in terms of an easier mourning process.

Finally, the need to investigate new and different approaches to witnessed resuscitation should be emphasized in the different areas in which nursing establishes its resuscitation work, from the perspective of the people who are experiencing this. Therefore, the use of the qualitative method should be boosted as a complement to the quantitative method, in order to understanding how the family’s presence during the resuscitation maneuvers is perceived.
